# Convolutional Fine-Tuned Threshold Adaboost approach for effectual content-based image retrieval

**DOI:** 10.1038/s41598-025-93309-6

**Published:** 2025-03-17

**Authors:** Robert Cep, Muniyandy Elangovan, Janjhyam Venkata Naga Ramesh, Mandeep Kaur Chohan, Amit Verma

**Affiliations:** 1 Department of Machining, Assembly and Engineering Metrology, Faculty of Mechanical Engineering, VSB-Technical University of Ostrava, 70800 Ostrava, Czech Republic; 2 Department of Biosciences, Saveetha School of Engineering, Saveetha Institute of Medical and Technical Sciences, Chennai, 602 105 India; 3 Applied Science Research Center, Applied Science Private University, Amman, Jordan; 4 Department of Computer Science and Engineering, Graphic Era Hill University, Dehradun, 248002 India; 5 Department of Computer Science and Engineering, Faculty of Engineering and Technology, Jain (Deemed-to-be) University, Bengaluru, Karnataka India; 6 Department of Computer Science and Engineering, Vivekananda Global University, Jaipur, India; 7 University Centre for Research and Development, Chandigarh University, Gharuan, Mohali, Punjab India; 8 Department of CSE, Graphic Era Deemed To Be University, Dehradun, 248002 India

**Keywords:** Content-based image retrieval (CBIR), High-level information, Deep and machine learning (DL, ML), Convolutional Fine-Tuned Threshold Adaboost (CFTAB), VGG-16, Computational science, Computer science

## Abstract

Applications for content-based image retrieval (CBIR) are found in a wide range of industries, including e-commerce, multimedia, and healthcare. CBIR is essential for organising and obtaining visual data from massive databases. Traditional techniques frequently fail to extract high-level, relevant information from images, producing retrieval results that are not ideal. This research introduces a novel Convolutional Fine-Tuned Threshold Adaboost (CFTAB) approach that integrates deep learning and machine learning techniques to enhance CBIR performance. This dataset comprises image-based data collected from multiple sources. This image data were pre-processed using Adaptive Histogram Equalization (AHE). The features of localized image data were extracted using VGG16. For an efficient CBIR process, a novel CFTAB approach was introduced. It combines both deep and machine learning (ML) methods in the proposed architecture to improve the excellence of image search. To further improve performance, CFTAB incorporates an improved AB algorithm. This algorithm adjusts the threshold levels dynamically within a robust classifier to optimize training outcomes.

## Introduction

Content-based image retrieval (CBIR) methods give assistance in classifying digital image archives based on their optical content. From an image similarity tool to a powerful annotating images engine, this notion states that CBIR encompasses^1^. The most popular kind of CBIR is visual search with image analysis. With the volume of digitally shaped images growing, new techniques for data archiving and access are essential. Textual searches on Metadata are possible in conservative databases. CBIR is a method that searches images from massive image databases in reply to user requests in the form of inquiry images by using visual content, recognized as features^2^. Edges can be used for image retrieval as they provide important information about an image. The spatial distribution of edges is captured by the edge histogram descriptor. Digital images created for scientific, educational, medical, industrial, and other purposes have become much more accessible in the recent several decades^3^. Managing the growing amount of visual data has become difficult. The field of CBIR has been developing quickly. CBIR employs visual material to conduct interest-based image searches across massive datasets. Visual information is extracted from an image by a conventional CBIR system, which then changes it internally into a feature with several dimensions depiction of vectors^4^. When computer-aided diagnosis involves some image-based processing, CBIR can be intriguing^5^. A trained system that does intelligent pre-selection of images might aid a physician in finding patients with comparable issues. An image’s visual content might be characterized by its color, texture, form, or spatial arrangement. A good description of visual information should be invariant to variations in light and insensitive to the particular imaging procedure^6^. The underlying color space must be defined before a color descriptor can be chosen. With the fields of computer vision and information retrieval, CBIR focuses on developing methods for obtaining images from big databases according to their visual content^7^. CBIR attempt to get well movies based on their graphical attributes including shape, color, texture, and spatial relationships, in contrast to conventional text-based retrieval systems and by focusing on the usefulness, precision, and usability of image repossession systems, Effective CBIR^8^. The adjective effectual denotes the capacity to bring about the intended effects. When it comes to CBIR, this entails creating retrieval system that match movies reliably based on visual characteristics while making the process user-friendly and efficient. Effective CBIR systems are necessary for many applications, such as multimedia satisfied management, observation, medical image analysis, and image hunt engines^9^. Through an efficacious approach, these systems aim to maximize retrieval speed and resource efficiency while offering consumers precise and significant findings. Effectual CBIR incorporate content-based image retrieval techniques with an emphasis on usability, effectiveness, and user happiness. It is essential for overcoming the difficulties in organize and locating images in big datasets from various fields^10^. The study’s preliminary retrieval results are obtained from images where considerable, high-level information is impossible to extract using current approaches. Moreover, the attempts made by CBIR systems to get better categorization performance hinder the development of effective methods to process the CBIR, we provide in this paper a novel CFTAB approach.

### The contributions of the study

- Retrieving relevant images using large-scale image collections repeatedly and consistently remains a difficulty in content-based image retrieval (CBIR).
- Images’ intensity had been significantly improved by preprocessing with Adaptive Histogram Equalization (AHE), which made key features simpler to identify and discriminate.
- Using VGG-16’s deep convolutional architecture, the study extracted high-level features from photos. The accuracy and efficacy of the content-based picture retrieval procedure were improved by the strong feature representations that this method offered.

The relevant study of the objective is shown in part 2. Collecting the dataset and processing the methodology are shown in part 3. In part 4 and 5, the performance analysis as well as discussion is presented and in part 6, the study is concluded.

### Problem statement

Effectively recovering relevant images from large-scale collections is a continuing difficulty in the field of CBIR. Current approaches frequently fail to extract high-level, relevant information from images, which results in less-than-ideal retrieval outcomes. Furthermore, the quest for enhancing classification performance impedes the advancement of effective methodologies in CBIR systems. To solve these problems, this paper suggests a novel method for CBIR termed Convolutional Fine-Tuned Threshold Adaboost (CFTAB). The goal of this strategy is to improve image search quality by combining deep learning and machine learning techniques. By using similarity index computations and model trials, the research shows that the CFTAB approach outperforms traditional CBIR approaches in conditions of obtaining identical images.

## Related works

The improved retrieval performance in single-class scenarios, but since various classes’ images had identical semantics, see a major decrease in performance in multi-class search contexts were examined in^11^. Due to highly connected semantic classes, CBIR techniques based on the hybrid classification model enhance retrieval accuracy as negative samples increase, but class imbalance biases classification toward the negative class. The drawbacks of the approach stated earlier in CBIR were the instability of the classification methods, mainly in multi-class environments where class imbalance is inherent, the classifying of features dominated in the negative class, and the need for further improvement in dealing with semantic similarities of highly correlated classes. Hybrid classifiers and genetic algorithms help in improving these cases. A cross-image Symmetric graph convolution networks (CIS-GCN) was used in^12^ to retrieve fine-grained diabetic retinal fundus pictures. The specific characteristics were then run through the compulsive localisation network to determine the lesion’s location characteristics. Disadvantages of their method relate to the challenges likely to arise in terms of generalizing from one patient population to another, limited scalability for larger datasets, dependence on quality fundus images, and validating the method across different clinical settings and imaging technologies. The proliferation of multimedia material online, CBIR systems, Keisham and Neelima^13^ suggested had emerged as a formidable obstacle for academics. The internet receives billions of images daily. It seems that the research community was facing a significant challenge while trying to find a relevant image on search engines. The CBIR system facilitated this search by providing feature vectors, which were high-level visual representations of images. The CBIR system include potential high computational complexity, dependency on accurate feature extraction, and challenges in handling diverse image types or large-scale databases. A-vascular Necrosis (AN) could cause muscular and skeletal disabilities proposed by^14^. It was common in young people, thus early diagnosis and treatment were required. This disease can produce femoral bone fractures that modify their structure. Common places were the ankles, lips, knees, and humerus. The varied fracture locations make AN-affected bone image difficult. The limitations of the proposed method include potential challenges with varying image quality, computational complexity, dependency on dataset diversity, and limited generalization to other medical conditions or image types. A novel approach to solving the CBIR problem was presented in the study^15^. A new Convolutional Neural Network (CNN) and a new framework for using RF have been considered to achieve this. It includes potential overfitting to benchmark datasets, reliance on specific feature extraction methods, and limited generalization to diverse real-world image retrieval scenarios or dynamic datasets. Efficient and successful CBIR was suggested^16^ examined Fuzzy c-means clustering, sparse complementary features for strong image representation along with locality-preserving projection for optimum, the method incorporates feature selection and uses a soft label support vector machine to classify strong images. The limitations of CBIR include the semantic gap between low-level features and high-level semantics, reliance on robust feature selection, and difficulty in handling diverse image content and varying datasets. Primary focus was on recent technical advances as they pertain to image representation and database search were indicated in^17^. Some limitations of proposed method include difficulty in handling large-scale information, complex image representations, variations in image quality, and the challenge of achieving high accuracy in diverse applications. Comprehensive system for retrieving images using a deep convolutional neural network (DCNN) presented in^18^ and a discrepancy knowledge approach. The proposed image retrieval system simplifies the retrieval process but may face challenges in scalability, generalization to diverse datasets, and handling large-scale real-time queries without compromising accuracy or efficiency. To restore the damaged parts of these images; the research suggested in^19^ a novel CBIR model that varieties use of an efficient mix of texture, form, and color data. Image scans were normalized and their noise was decreased using a median filter for this purpose. The limitations of the proposed Content-Based Image Retrieval model include potential inaccuracies in reconstructing highly corrupted regions, reliance on pre-processing steps, and possible challenges with generalizing to diverse image types. Feature extraction and selection that affects CBIR and involves data extraction from images by use of both global and local as well as several feature extraction along with selection methods were examined in^20^ to extract the best features for CBIR. Some limitations of CBIR include difficulty in handling complex and varied images, dependence on feature selection, limited accuracy in retrieving highly similar images, and challenges with scalability and real-time performance. Based on approximate homomorphic encryption (HE) and CBIR, Wang et al.^21^ suggested a safe and effective ciphertext image retrieval method. After resizing the photographs, we first encrypted them using approximate homomorphic encryption. Then, it uploaded the ciphertext images to the cloud so that the ciphertext’s features could be extracted. Limitations include potential data inflation from homomorphic encryption, increased computational costs due to large image sizes, and challenges in maintaining efficiency while ensuring secure, accurate retrieval of encrypted images. The research^22^ examined must be closed for effective retrieval, which is the primary issue with CBIR systems. The diagnosis of malignant lung nodules and several other lung disorders was aided by the common imaging signals (CISs) that show up on the patient’s lung CT scan. The work provides a novel set of descriptors for the successful recovery of these imaging indicators. The limitations of the proposed system include potential challenges in reducing the semantic gap completely, dependency on accurate feature selection, and the need for large, diverse datasets to ensure robust retrieval performance. Automatically generated image attributes including color, texture, and form, CBIR was a method for recovering medical images suggested in^23^. CBIR has several uses, including electronic patient records, research, diagnosis, and education. Researchers have spent decades studying the encoding of image characteristics, which was largely responsible for a CBIR system’s retrieval performance. The reported CBIR technique’s drawbacks include its inability to bridge the conceptual gap among high-level ideas and low-level image data and its reliance on the selected similarity factors for retrieval accuracy. CBIR with a convolutional Siamese neural network (CBIR-CSNN) is the method proposed in^24^. The datasets were created by cropping off the lesion patches, and a patch-pair dataset was created by pairing two arbitrary patches. Second, a CSNN is trained using this patch-pair dataset. The limitations include potential challenges in generalizing to larger, more diverse datasets, the reliance on patch-level analysis, and reduced performance on the external dataset compared to the primary dataset. Recovering comparable images; the paper suggested^25^ a definitive CBIR strategy that integrates visual and linguistic characteristics. The method begins by classifying the query image as either textual or non-textual. The query image was categorized as textual if there was any text inside it. This approach include potential challenges in accurately classifying textual and non-textual images, the complexity of feature fusion, and possible inefficiency with diverse or noisy datasets. On the basis of lightweight neural network models, Zhang et al.^26^ suggested a novel offline retrieval paradigm. By reconstructing the network topology, adding the convolutional attention module, and adding pointwise group convolution and channel shuffling into the bottleneck block, it concentrates on the model’s feature extraction in this framework. The limitations for content-based image retrieval include high computational and memory requirements, redundancy in lightweight models, and lack of consideration for terminal-based deep learning image retrieval applications. Provided two-stream attentive CNNs with saliency incorporated into CBIR, motivated^27^ by the results. Two streams in the suggested network manage two jobs at once. The major emphasis was on extracting features from images that were discriminative and closely associated with semantic properties. The limitations of this approach include challenges in accurately capturing user query intention, the variability of saliency across different models, and potential over-reliance on visual saliency in diverse contexts. The research^28^ offered an image retrieval approach that used constraint metrics to create human gaze-shifting routes based on saliency maps, therefore narrowing the semantic gap in image retrieval. The limitations of the proposed method include dependency on high-quality salient regions, complexity of designing constraint metrics, and potential challenges in generalizing across diverse image datasets and domains. By dividing the data, Yenigalla et al.^29^ used databases to investigate the CBIR system utilizing three ML techniques. Additionally, evaluate the accuracy and efficiency of each algorithm when it is applied to a particular image retrieval task. Limitations of CBIR include computational complexity, difficulty in handling diverse image types, limited ability to interpret semantic meaning, and challenges in scaling for large, varied multimedia databases.

To filter and minimise the images that were different to the query image in the image collection, Salih and Abdulla^30^ created a two-layer based CBIR technique. Because the bag of feature (BoF) was applied in the first level, the most comparable images were saved for the following layer. In between, the most dissimilar images were removed, it reduced the search area for the following stage. Concatenating the retrieved texture and color-based information was the primary focus of the second layer. The limitations of their approach include dependency on local features for initial retrieval, potential inefficiency in handling large datasets, and limited scalability of feature extraction methods across diverse image types.

A novel CBIR approach involving two layers, referred to as bi-layers, had been evaluated in^31^. The M nearest images to the querying image were obtained in the first level after all database images were associated to it using the BoF approach. The second layer retrieved the images that were maximum comparable to the querying image by comparing the M images from the first level to it using colour, texture, and form attributes. Limitations of the proposed bi-layer CBIR technique include reliance on feature extraction accuracy, computational complexity, and potential inefficiency with larger datasets or more diverse image types.

### Problem statement

Image retrieval based on content and other image attributes, such as pixel index matching, is necessary. Clustering methods, which use a subset and superset properties approach, are necessary for grouping similar qualities. To do similarity matching and mapping, three different levels of thresholding are required: high, low, and average. In the case of complicated traits and properties, evolutionary algorithms might be crucial. To facilitate effective retrieval, a combination of data filtering, grouping, and threshold value mapping is required. Dimension and magnitude variables need algorithms. The use of adaptive thresholding based on pixel indices improves image retrieval in our CFTAB approach. It handles complicated qualities by improving feature extraction using CNN. Reliable similarity matching was guaranteed by adjustable thresholds at high, low, and medium levels. Overcoming difficulties in image attribute matching; this all-encompassing method improves data filtering, grouping, and threshold mapping for efficient retrieval.

## Methodology

**Fig. 1:**
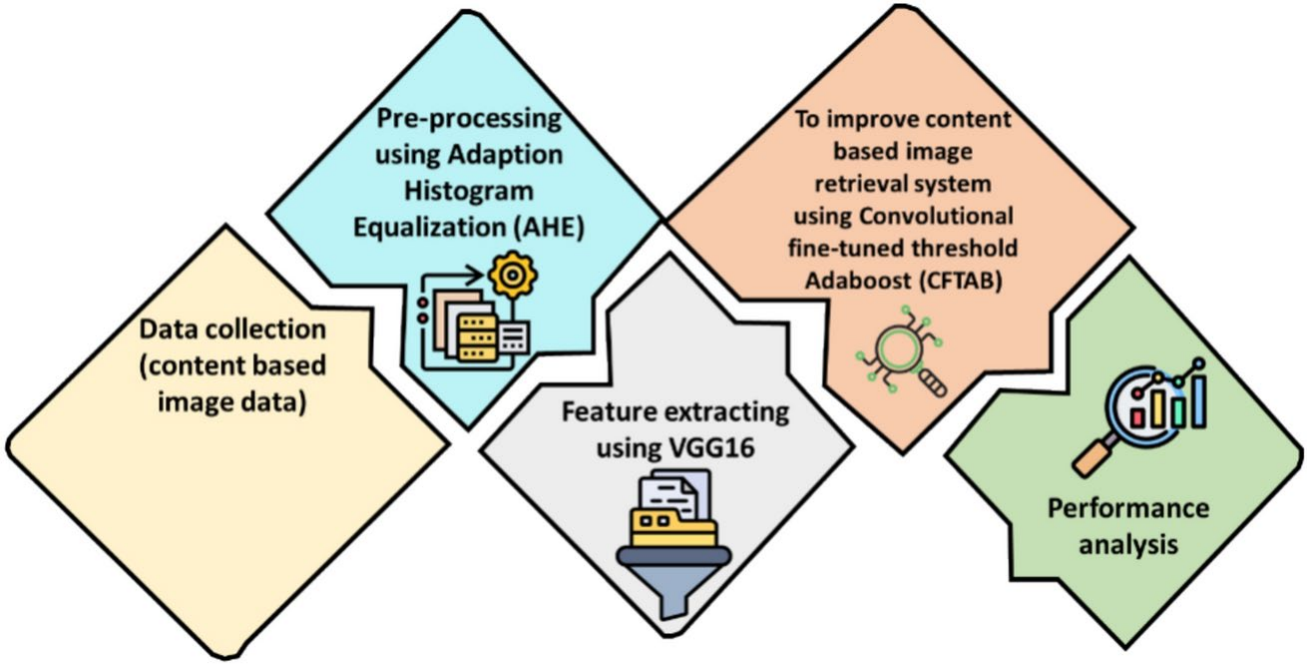
Flow of proposed.

The purpose of this study was to extract features and apply methods utilizing a CBIR dataset. A wide variety of visual information was included in the collection. This image data were pre-processed using Adaptive Histogram Equalization (AHE). This localized image data were extracted using VGG16. To analyze the CBIR process using Convolutional Fine-Tuned Threshold Adaboost (CFTAB), for to get better categorization performance hinders the development of effective methods. The flow of the CBIR-based methodology is shown in Fig. 1.

### Data collection

In this study, we collected the dataset from a Kaggle source. Two types of data were collected for this investigation.

*Dataset 1*: As with the majority of vision-related tasks, CBIR has been dominated by DL models in the last decade. Nevertheless, the majority of articles that discuss ways to improve neural networks for CBIR use datasets that are relevant to the domain to train and evaluate their models. Consequently, whether or not such networks are suitable for use as a general-purpose feature extractor for images is not apparent. We chose to create GPR1200, a hard benchmark dataset containing 1200 categories and 10 class samples, after studying popular image retrieval test sets. GPR1200 is straightforward to use and accessible. To guarantee high-class variety and crisp class borders, six publicly accessible datasets covering various image regions were used for manual class and image selection (https://www.kaggle.com/datasets/mathurinache/gpr1200-dataset).

*Dataset 2*: For image search utilizing Pet Dataset (https://www.kaggle.com/datasets/gunarakulangr/sri-lankan-wild-elephant-dataset).More than 10,000 photos documenting different facets of Asian wild elephants in their natural environment make up the dataset. For a wide range of possible artificial intelligence (AI) applications, these pictures are an invaluable resource. This dataset provides a comprehensive and diverse set of visual information about Asian wild elephants. The extensive dataset enables a wide range of AI applications, including elephant identification, alert detection for elephant assault, behavioural analysis of elephants, and more. YouTube recordings of Asian wild elephants were sourced as part of the data collecting procedure. A large picture dataset was then produced by using a specific algorithm to extract individual frames from these films. This method guaranteed a thorough and organized gathering of visual data, supporting a range of research projects and AI.

### Data pre-processing Adaptive Histogram Equalization (AHE)

**Fig. 2:**
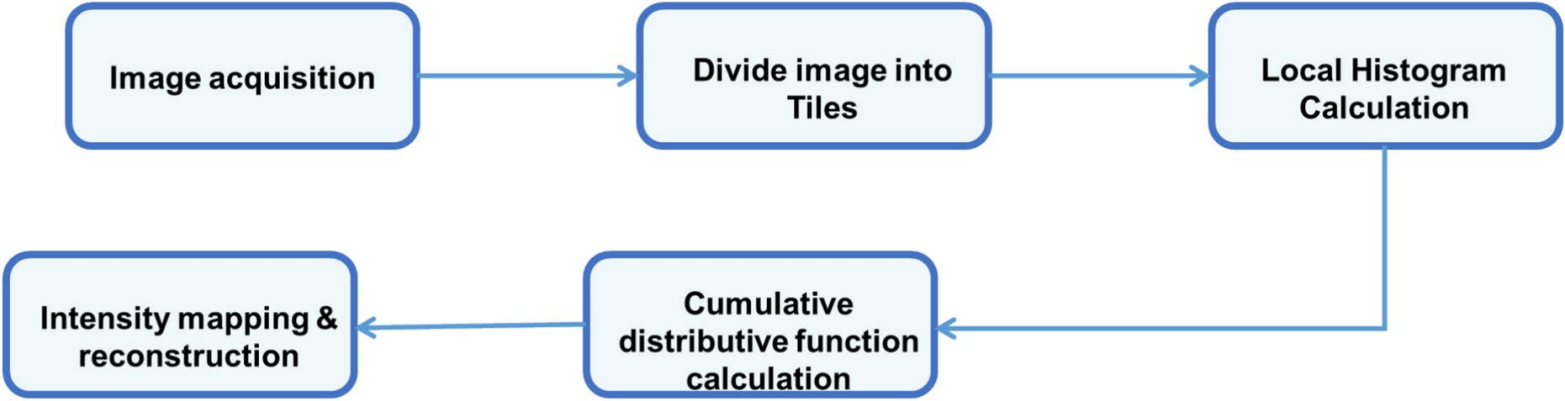
Process of AHE.

After collecting the image data, AHE is used for preprocessing. By dispersing pixel intensity levels according to local histograms, it is used to improve image contrast. Important details become easier to see as a result of the enhanced visibility of elements in both bright and dark areas. Applying AHE improves performance in content-based picture retrieval tasks by increasing the efficacy of subsequent feature extraction procedures. Figure 2 represents the AHE process. This method assigns a contextual region, which is a region centered on the pixel’s neighborhood, to each pixel in the image. The pixels are next subjected to histogram equalization mapping, which is determined by the average intensity values in that area. The following notations are used to accomplish histogram mapping. If $(w,z)$ is an intensity $j$ pixel from the image.

$${n^\wedge} (+-) \; the \;mapping \;of \;right\; upper\; {w^\wedge}(+-)$$

$${n^\wedge} (++)+\; the\; mapping \;of \;right \;lower \;w^\wedge (++)$$

$$n^\wedge(-+) \; the \;mapping \;of \;left \;upper \;w^\wedge(-+ ) \;and$$

$$n^\wedge(--) \;the \;mapping \;of \;left \;lower \;w^\wedge(-- )$$

The output enhanced image is then created by combining the nearby sections using bilinear interpolation to remove artificially created boundaries. The following Eq. (1) is used to calculate the interpolated adaptive histogram equalization coefficient:

1 $$N\left(s\right)=\alpha \left[a,{n}^{--\left(s\right)+\left(1-a\right).{n}^{+-}}\left(s\right)\right]+\left(1-b\right).[a.{n}^{-+}\left(s\right)+\left(1-a\right).{n}^{-+}\left(k\right)]$$

where,

$$b=(z-{z}^{-})/({z}^{+}-{z}^{-})$$

$$a=(w-{w}^{-})/({w}^{+}-{w}^{-})$$

AHE tends to overamplify noise, especially in homogenous areas, enhancing the image to the point where things that weren’t there or visible in the original image are created.

### Feature extraction using VGG16

After the pre-processing, localized image data were extracted by VGG16. Using its deep convolutional architecture, which captures spatial hierarchies, VGG-16 is used in this stage to extract high-level visual information from the images. Patterns, textures, and shapes that are essential for differentiating between various image categories can be found with the use of VGG-16. The resultant feature vectors will help the content-based image retrieval system compare and retrieve images more efficiently. Train and test sets are created from the datasets. One of the most popular DL architectures, VGG16, was introduced by Oxford University. Its 41 constituent layers are shattered in this way: In all, there are thirty-three FC layers, sixteen weight layers, and thirteen convolutional layers (Conv.). With a stride of one, VGG16 employs a small three-by-three kernel (filter) on every Conv. Layer. In every case, the Max pooling layers are before the Conv. Layers. The input images to VGG16 have a fixed resolution of 224 × 224 and three channels. Every one of VGG16’s three FC levels has its unique depth. The first two shares a channel size of 4096, but the final FC sports a channel size of 1000, symbolizing the number of class labels found in the image dataset. The output is the responsibility of the soft-max layer, which is responsible for the input image’s given probability. Randomly initializing the weights in VGG16, like any pre-trained model, requires extensive training. In other words, mimic the input and output of the current situation as much as possible while training a CNN model. Here, train the VGG 16 model using the ImageNet dataset, which has many images of objects in their natural habitat. The CNN framework VGG-16 is renowned for being both user-friendly and stylish.

Interestingly, after employing small 3 × 3 convolutional filters with an unchanged stride of 1 pixel, it employs max-pooling layers for spatial downsampling. Each convolutional layer is followed by a repaired linear unit (ReLU) activation function to enhance feature extraction efficiency. The network is quite good at activities including image categorisation and extraction of features because of its deep structure, which allows it to extract complex sequences from images. VGG-16 is still a popular option in machine vision as it continuously yields cutting-edge results on a variety of benchmarks. This extracted data will be sent to the CBIR process will receive the feature vectors that were extracted from the image data. To retrieve pertinent images in response to user queries or input images, the feature vectors will be used in this stage to compare and match images according to their visual content.

### Content-based image retrieval (CBIR) using Convolutional Fine-Tuned Threshold Adaboost (CFTAB)

CFTAB is used by CBIR to achieve accurate image matching. Convolutional neural networks (CNNs) and optimized Adaboost classifiers are used in this sophisticated method to provide excellent feature extraction and precise thresholding. CBIR with CFTAB provides effective image retrieval and improves retrieval relevance and accuracy for a variety of applications.

#### Convolutional neural network (CNN)

**Fig. 3:**
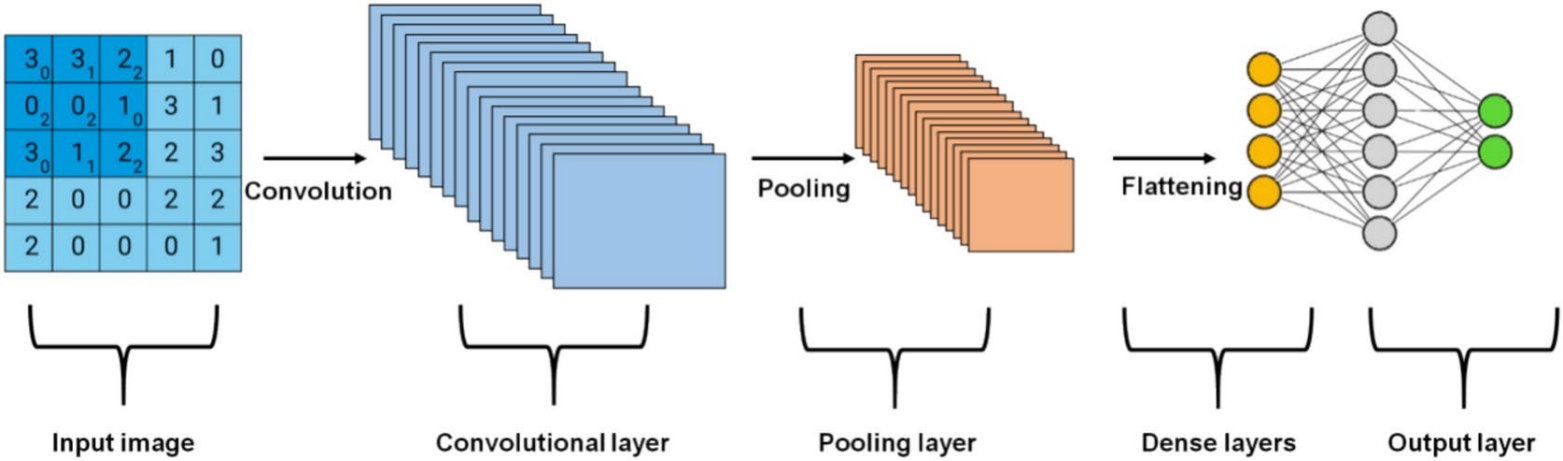
Architecture of CNN.

CNN is a class of neural networks that draw inspiration from biological processes. Their discoveries have led to good results in a wide variety of machine-learning tasks. Therefore, CNNs are regarded to be feed-forward systems because information moves in a single direction from inputs to outputs. Biological notions are essential to the functioning of the CNNs. Layers of basic and sophisticated cells alternate in the brain’s visual cortex and they are responsible for determining the arrangement of these structures. Even though there is a broad range of CNN designs, they are structure-based and comprised of CNN as well as pooling layers. Following these components are one or more layers that are entirely coupled to one another and have an architecture that is similar to that of a feed-forward neural network. To construct a deep design, components are stacked on top of one another. Figure 3 is a graphical representation of the overall framework of a CNN, including its principal parts such as the input, convolutional, pooling, and flattening layers; this procedure involves integrating data into a hierarchical structure of dense layers, which stands for the outcome obtained in the output layer.

For CNNs, dealing with massive amounts of training data requires a mini-batch technique. Let $Y=\left[{y}_{1},\dots .{y}_{N}\right]$ consists of activation features from a set of N training images, where the dimensionality of the feature vector ${y}_{j}$ is $R$ and $\left[{y}_{1},\dots .{y}_{N}\right],\in \{-\text{1,1}\}$ saves the ground truth labels in a vector. A powerful classifier determines the forecast using the boosting technique. $Z(\cdot )$ is the total of all the weak classifiers weighted together $z(\cdot )$ as follows:

2 $$Z\left({y}_{1}\right)=\sum_{i=1}^{R}{\alpha }_{i}z\left({y}_{ji},{\lambda }_{i}\right);$$

3 $$z\left({y}_{ji},{\lambda }_{i}\right)=\frac{l\left({y}_{ji},{\lambda }_{i}\right)}{\sqrt{l{\left({y}_{ji},{\lambda }_{i}\right)}^{2}+{\eta }^{2}}}$$

where, ${y}_{ji}\epsilon {y}_{j}$ xi is the ${i}^{th}$ activation feature of the ${j}^{th}$ image. A potential poor classifier is associated with each attribute $z\left({y}_{ji},{\lambda }_{i}\right)$ with output in the range of ($-\text{1,1}). \frac{l(.)}{{l(.)}^{2}+{\eta }^{2}}$ is used to simulate a sign $(\cdot )$ method for optimizing gradient descent via derivative computation has been developed. In this work, $z\left({y}_{ji},{\lambda }_{i}\right)\in {\mathbb{K}}$ has the criterion for considering a decision stump, which is a one-level decision tree, and ${\lambda }_{i}$ , which AdaBoost (AB) has made extensive use of. The parameter $\eta$ to manage the function’s ramp $\frac{l(.)}{\sqrt{{l(.)}^{2}+{\eta }^{2}}}$ as well is adjustable by the distribution of $l(\cdot )$ as $=\frac{\sigma }{v}$ , where $\sigma$ is the standard deviation of $l(\cdot )$ and $c$ is a constant. In this work, $\eta$ is empirically set to $\frac{\sigma }{v}$ . ${\alpha }_{i}\ge 0$ is the weight of the ${j}^{th}$ weak classifier.

4 $${\sum }_{i=1}^{R}{\alpha }_{i}=1$$

When ${\alpha }_{i}=0$ because it is dormant, the associated neuron will not engage in feed-forward or back propagation. This formula represents a summation notation. It states that the sum of ${\alpha }_{i}$ for $i$ ranging from 1 to $R$ equals 1. In simpler terms, it means that when you add up all the values of ${\alpha }_{i}$ from 1 to $R$, the total sum is equal to 1.

5 $${\varepsilon }^{P}={\beta \varepsilon }_{storng}^{P}+(1-\beta ){\varepsilon }_{weak}$$

where, $\beta \in [0, 1]$ strikes a balance between the losses experienced by strong and weak classifiers.

6 $${\varepsilon }_{storng}^{P}=\frac{1}{M}\sum_{j=1}^{M}(Z\left({y}_{j}\right)-{y}_{j}{)}^{2}$$

7 $${\varepsilon }_{weak}=\frac{1}{NM}\sum_{j=1}^{M}\sum_{\begin{array}{c}1\le i\le R\\ {\alpha }_{i}=0\end{array}}[z\left({y}_{ji},{\lambda }_{i}\right)-{x}_{j}{]}^{2}$$

where the constraint ${\alpha }_{i}> 0$ does not include dormant neurons in the loss calculation.

#### Threshold Adaboost (FTAB)

An overview of the multi-class classification issue and the TAB is provided before getting into the new multi-class boosting approach. Assume that having access to some training data $({y}_{1},{v}_{1}),...,({y}_{m},{v}_{m})$, where the input ${y}_{j}\in {\mathbb{K}}^{b}$ and the output ci have a subjective nature and it is assigned a limited number of values, e.g. $\{1, 2,..., R\}$. K is the sum of all the classes. It is assumed that the training data are independent and identically distributed samples from a random distribution Prob $(Y, V)$. The objective is to provide a rule for categorization $(y)$ based on the data used for training, allowing us to assign a class label c to fresh input y $\{1,..., R\}$. Under the $0/1$ loss, this is the formula for the classifier’s misclassification error rate:

8 $$1-{\sum }_{r=1}^{R}{A}_{Y}\left[{\mathbb{j}}_{V\left(Y\right)=r}Prob(V-r|Y)]\right]$$

It is clear that,

9 $${V}^{*}\left(y\right)=\text{arg}\begin{array}{c}max\\ r\end{array}Prob(V-r|Y=y)$$

To reduce this amount while keeping the misclassification error rate at,

10 $$1-{A}_{Y}{max}_{r}Prob(V-r|Y)$$

The error rate of this classifier is called the Bayes error rate and it is called the Bayes classifier. Nearest Bayes classifier approximation is the goal of the iterative FTAB method ${V}^{*}(y)$ using a combination of many underperforming classifiers. The AB constructs a classifier, such as a classification tree that generates labels for classes, using the un-weighted training data. Training data points are given more weight (boosted) if they are misclassified. With the new, unequal weights, a second classifier is constructed. Enhance the weights of the misclassified training data again and do it all over again. This is a common method for creating 500 or 1000 classifiers. Every classifier gets a score and the final classifier is the linear combination of all the classifiers from each step. The first step of the FTAB method is as follows, where (*y*) is a weak multi-class classifier that labels *y* with a class. The FTAM Algorithm is shown in Algorithm 1.

There is no better off-the-shelf classifier than AB with trees and it has been shown that AB. This does not hold for issues involving several classes, even though AB was suggested for use in such a scenario. Theoretically, weak classifiers are predicted to have an error rate less than $1/2$, about the training distribution. In a multi-class setting, when the probability of a mistake due to random guessing is higher, this is much more difficult to do $(R-1)/R$. The creators of AB noted that their inabilities to deal with poor learners whose mistake rates are higher than 1/2 is the algorithm’s primary drawback. Therefore, AB can struggle in a multi-class setting. Here takes a look at a basic three-class simulation to show what means. Each input $y\epsilon {\mathbb{K}}^{10}$ and a ten-dimensional standard normal distribution are used to choose the ten input variables for each training sample. Here are the definitions of the three classes:

11 $$v=\left\{\begin{array}{ll}1, & \quad if \;0\le \sum {y}_{i}^{2}<{\chi }_{10,1/3,}^{2}\\ 2, & \quad if\; {\chi }_{10,1/3,}^{2}\le \sum {y}_{i}^{2}<{\chi }_{10,1/3,}^{2}\\ 3,& \quad if \; {\chi }_{10,1/3,}^{2}\le \sum {y}_{i}^{2}\end{array}\right.$$

**Algorithm 1:**
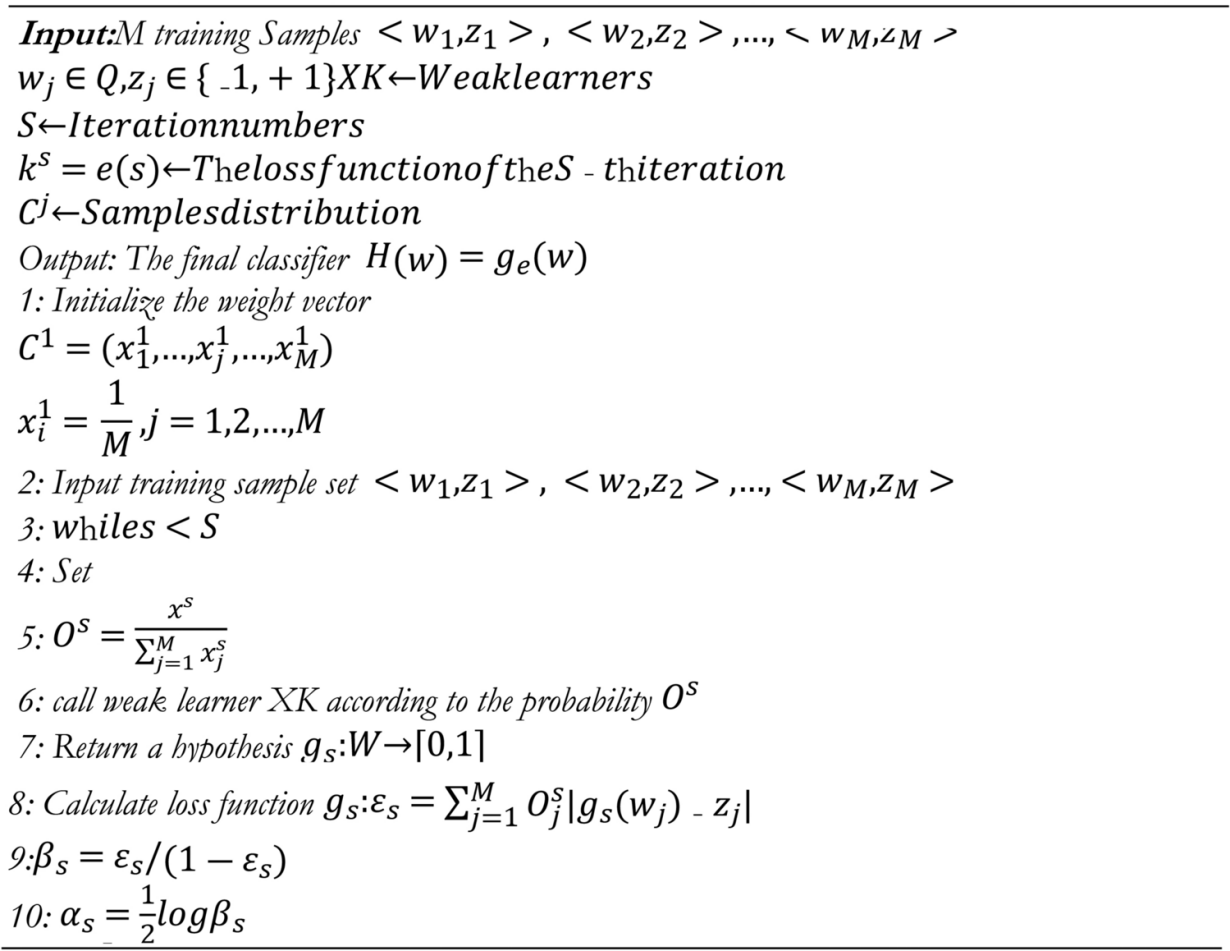
FTAB algorithm.

## Results and discussion

**Fig. 4:**
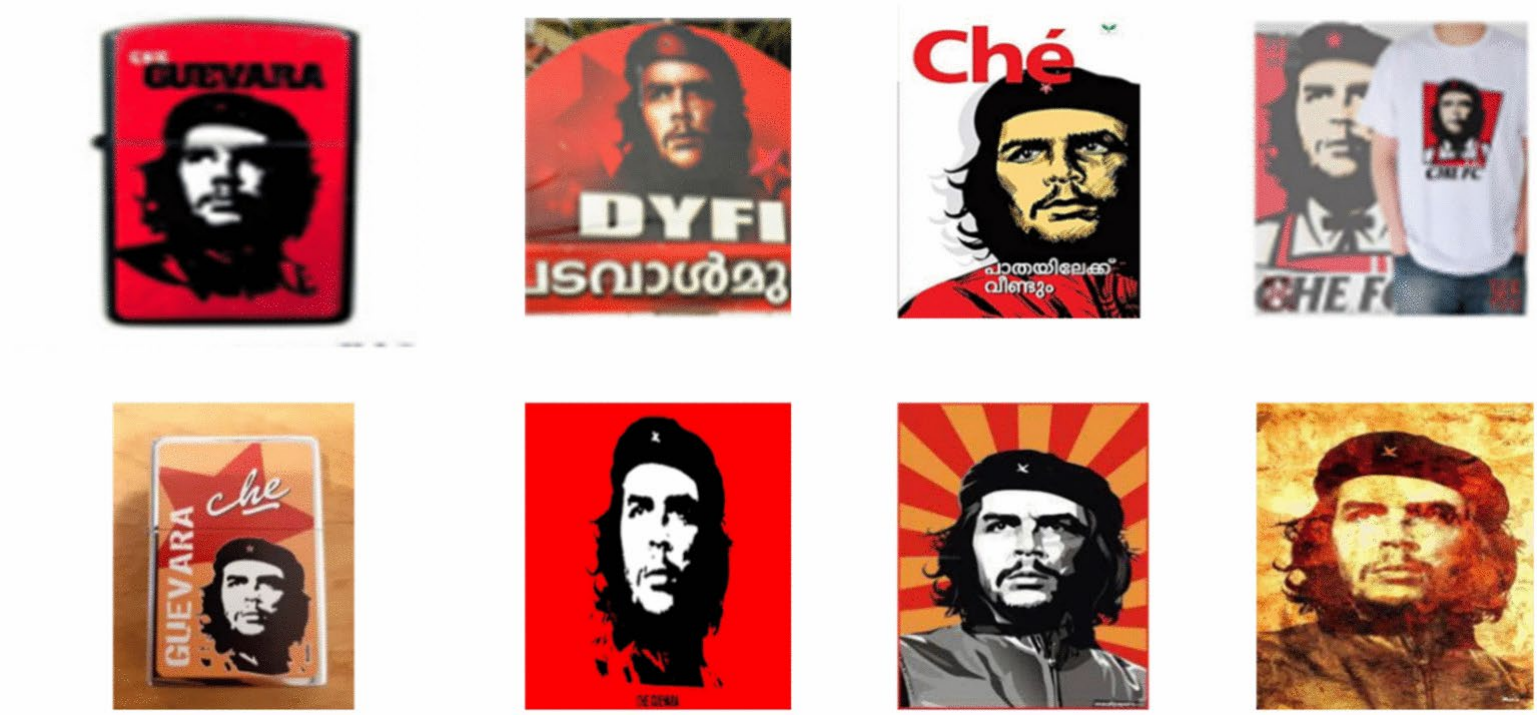
Image retrieval with CFTAB (Dataset 1).

**Fig. 5:**
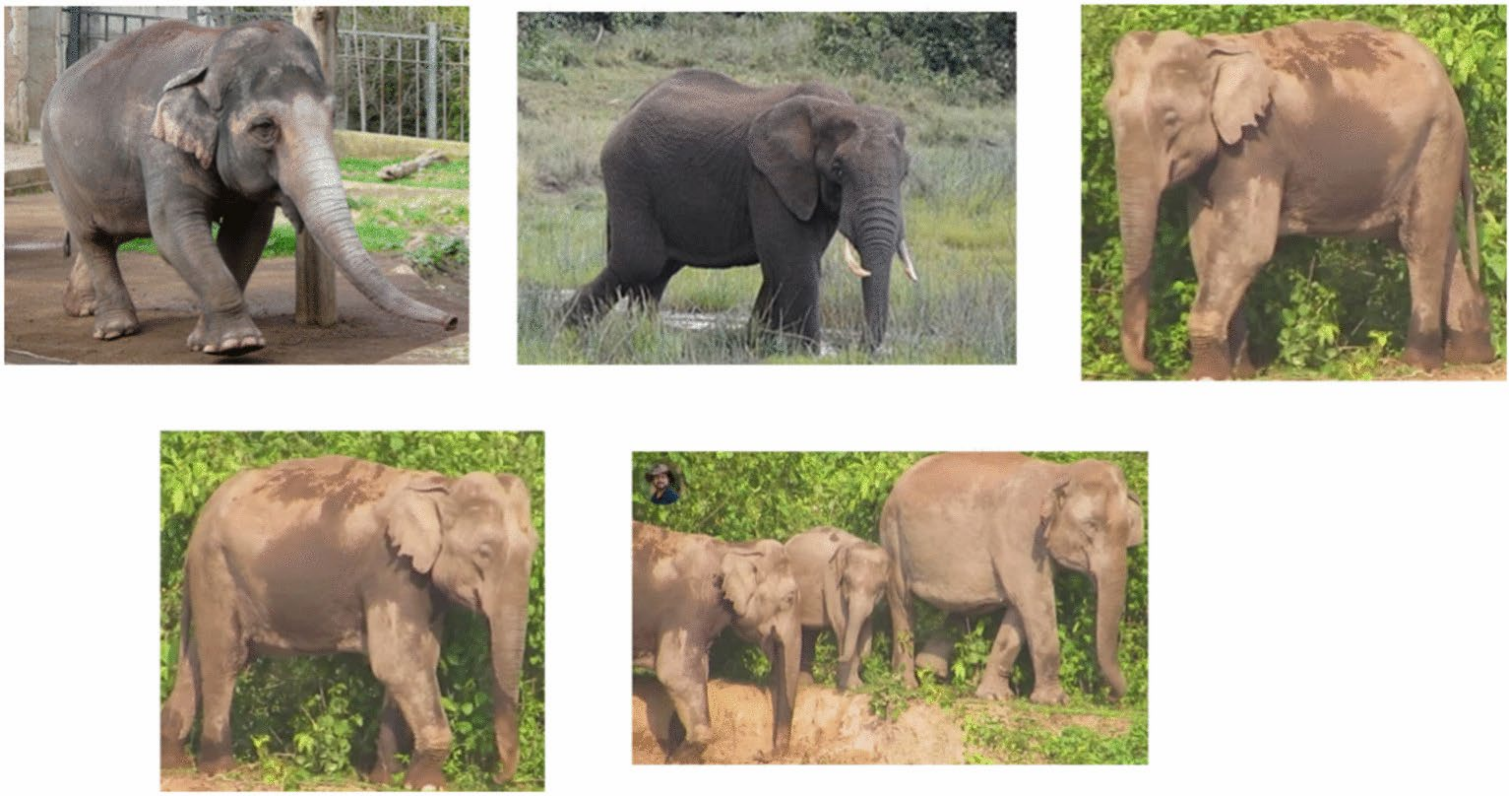
Image retrieval with CFTAB (Dataset 2).

We utilize Python 3.8 on Anaconda and the open-source Tensorflow-Keras package from Google for training. The laptop’s graphics are enhanced with Intel^®^ Iris^®^ Xe and it runs on a powerful 11th Gen Intel^®^ CoreTM i7-1165G7 CPU @ 2.80 GHz. With its massive 512 GB disk space and massive 16 GB RAM, it handles multitasking. Using CBIR-based CFTAB methods, this research analyzed data. The error rate, f1-score, recall, accuracy, and precision are some of the statistics used to measure a model’s predictive power in CBIR classification tasks and compared with existing techniques such as Rotation-invariant uniform Local binary patterns (RULBP)^32^, Xception^33^, MobileNet^33^, Inception^33^, Gray level co-occurrence matrix (GLCM)^32^, DL based enhanced convolutional neural network (GLCM-DLECNN)^32^. Image Retrieval outcomes are depicted in Figs. 4 and 5.

### Accuracy loss

**Fig. 6:**
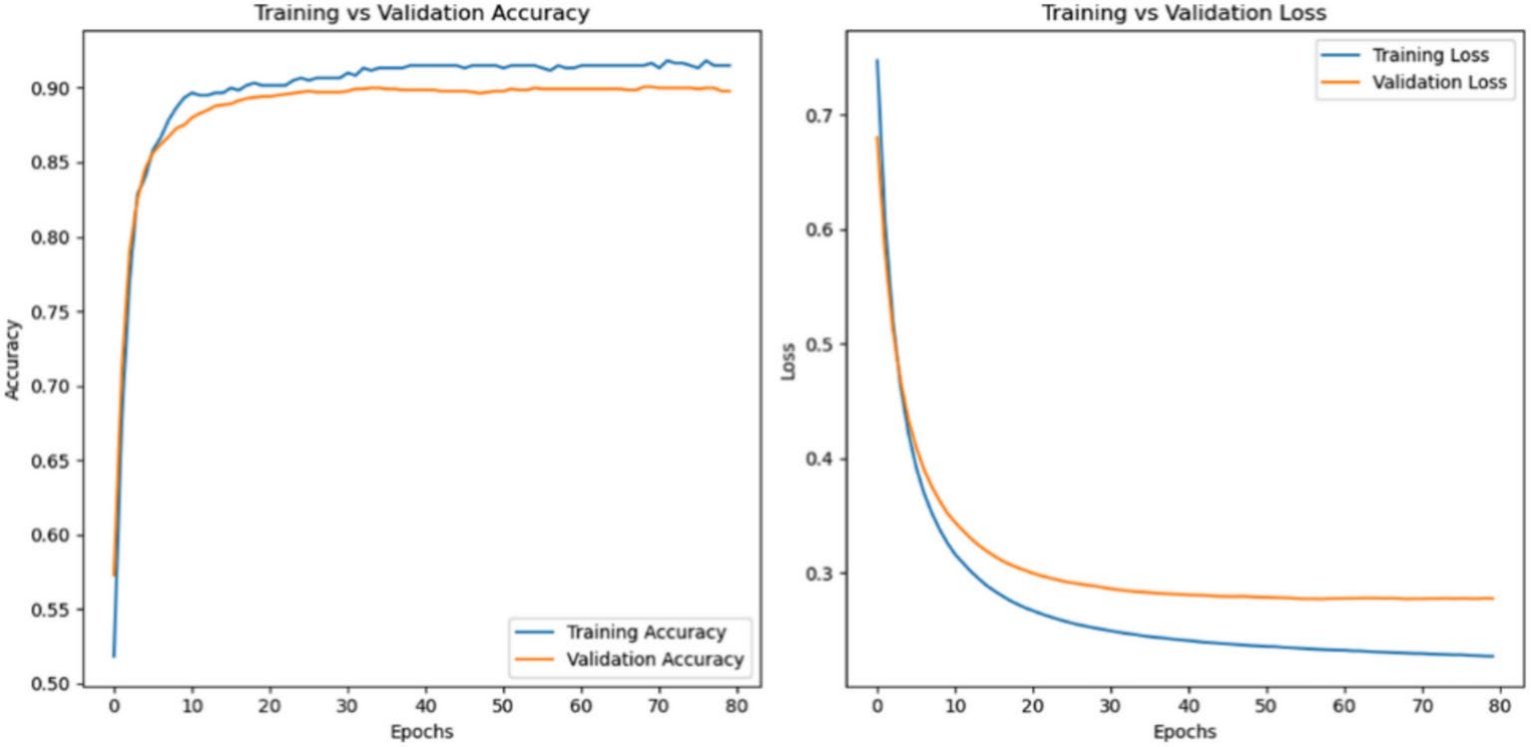
Accuracy loss outcome.

A performance metrics for training and validation throughout 80 epochs. Accuracy is displayed in the left plot, where training and evaluation accuracy both rise and level off towards the end, signifying successful model learning. The right plot shows the loss, with training loss decreasing faster than validation loss, which might suggest some degree of overfitting. This visual can be used to assess the model’s generalization ability, which is crucial for effectual Content-Based Image Retrieval tasks. Figure 6 presents the accuracy loss.

### ROC curve

**Fig. 7:**
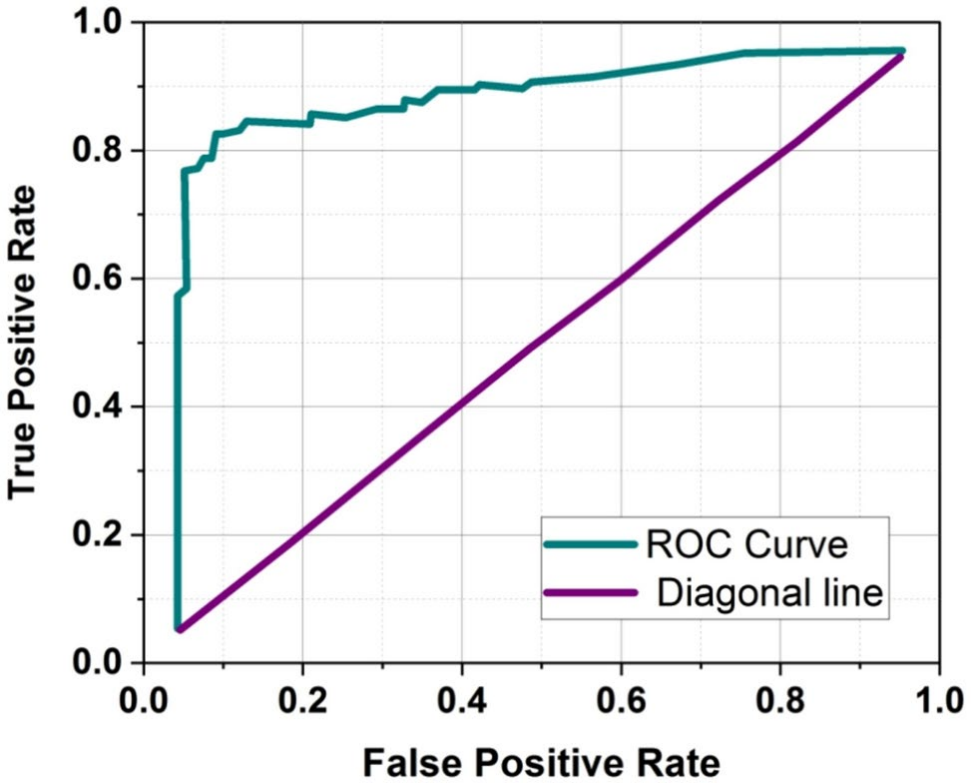
ROC curve.

A Receiver Operational Characteristics (ROC) curve is a graphic representation of a binary classification system’s capacity to identify issues when its distinguishing threshold is altered. The graph is produced by comparing both True Positive Rates (TPR) and False Positive Rates (FPR) at various threshold settings. This is the ROC curve in Fig. 7.

### Accuracy

**Fig. 8:**
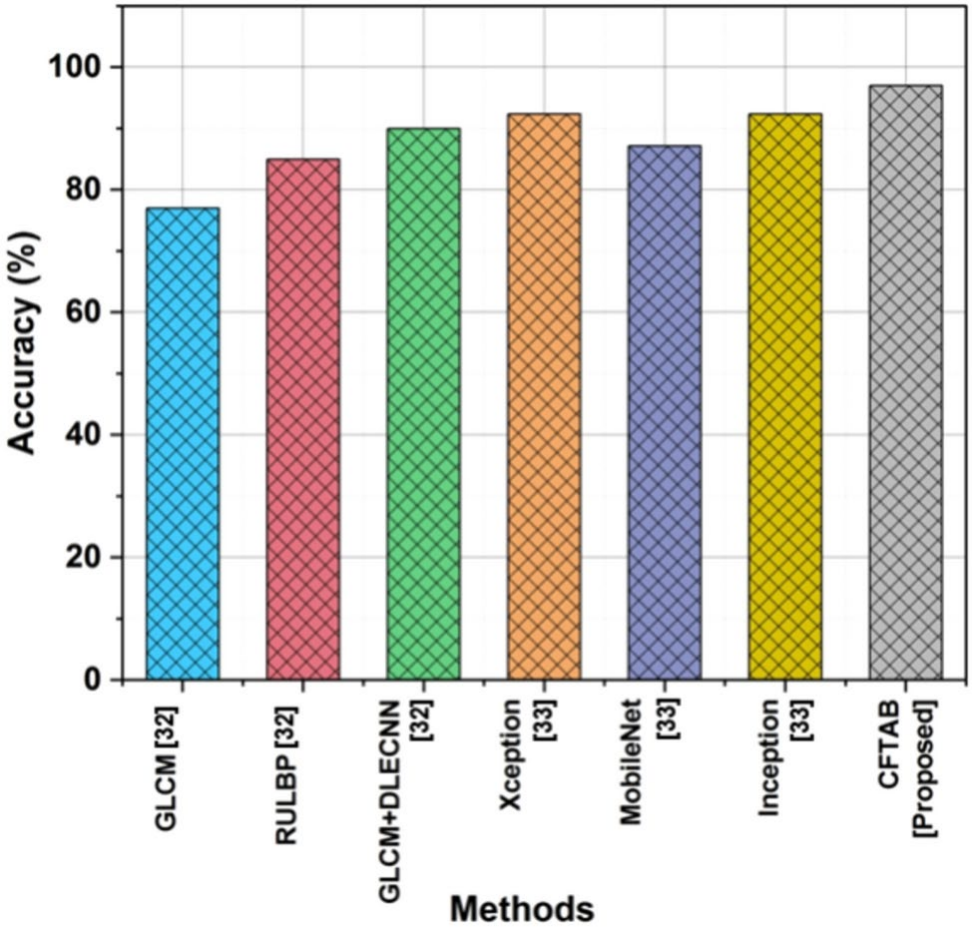
Accuracy.

The efficiency and accuracy with which a CBIR organization can repossess appropriate images based on their visual content is referred to as CBIR accuracy. The ability of a CBIR system to accurately identify and rank images that resemble the query image in terms of visual content is a key indicator of its accuracy. To provide consumers with the most relevant results, this entails limiting false positives, which retrieve irrelevant images, and false negatives, which ignore useful images. Table 1 and Fig. 8 show the accuracy of CBIR.

**Table 1:** Outcome of accuracy.

Methods	Accuracy (%)
GLCM^32^	77
RULBP^32^	85
GLCM + DLECNN^32^	90
Xception^33^	92.375
MobileNet^33^	87.125
Inception^33^	92.375
CFTAB [proposed]	97

12 $$Accuracy=\frac{Tp+Tn}{Tp+Tn+Fp+Fn}$$

In image analysis and retrieval tasks, the suggested approach, CFTAB, shows an outstanding 97% accuracy, surpassing state-of-the-art methods like GLCM (77%), RULBP (85%), GLCM + DLECNN (90%), Xception(92.375%), MobileNet (87.125%),Inception (92.375%) and proving its effectiveness in improving accuracy and performance.

### Precision

**Fig. 9:**
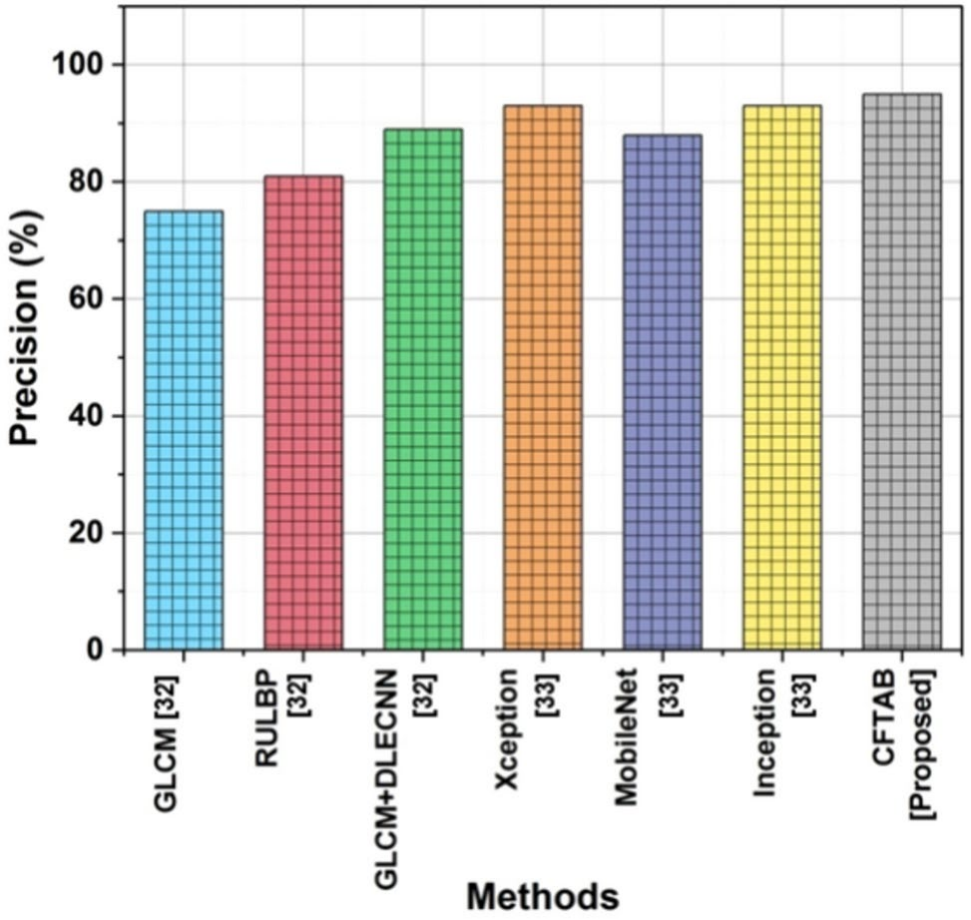
Precision.

The precision of CBIR is measured by how well it can respond to user queries by retrieving appropriate images from a database. Precision quantifies the percentage of images that meet the user’s information demands. Precision measures a CBIR system’s ability to avoid returning irrelevant images to a user’s query. A greater precision score indicates that the system retrieves images that closely match the user’s requirements, improving retrieval precision and satisfaction. Table 2 and Fig. 9 show the precision of CBIR.

**Table 2:** Analysis of precision.

Methods	Precision (%)
GLCM^32^	75
RULBP^32^	81
GLCM + DLECNN^32^	89
Xception^33^	93
MobileNet^33^	88
Inception^33^	93
CFTAB [proposed]	95

13 $$Precision=\frac{Tp}{Tp+Fp}$$

In the area of image retrieval or classification, the suggested approach, CFTAB, performs better than GLCM, RULBP, and GLCM + DLECNN, which obtained precisions of 75%, 81%, and 89%,93%,88%,93 respectively, as well as CFTAB above current methods with a precision of 95%.

### Recall

**Fig. 10:**
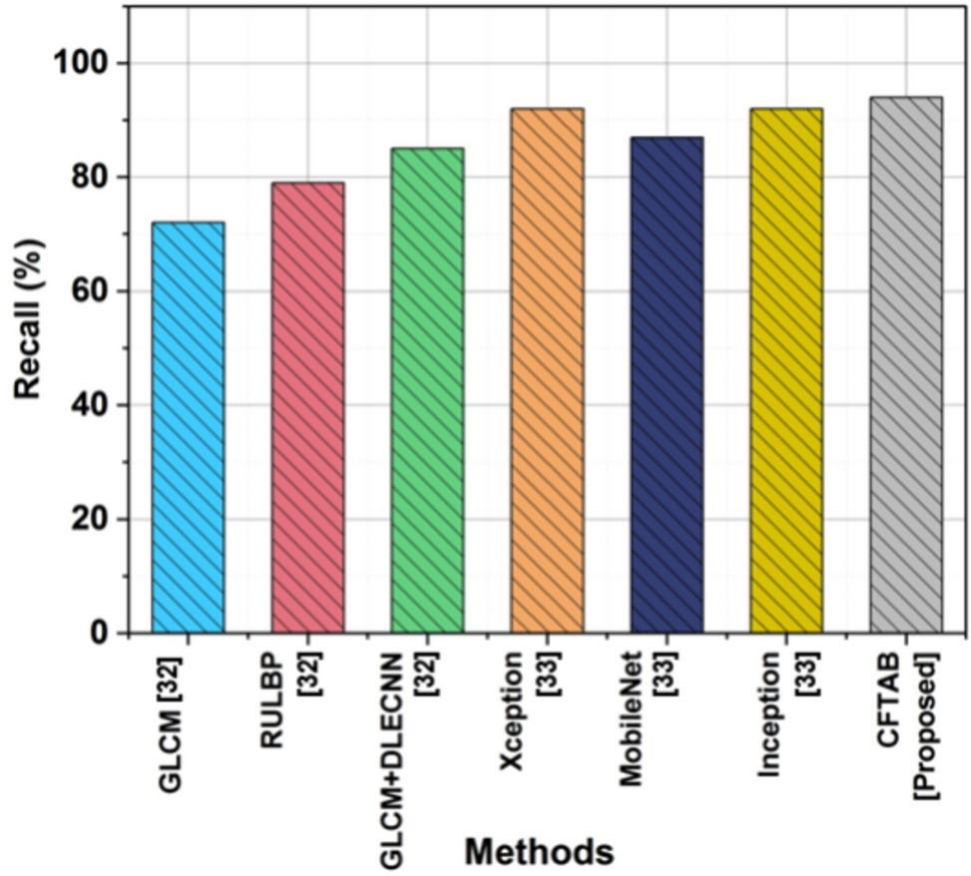
Recall.

When discussing CBIR, the term recall refers to the capability of a system to recover the relevant images found in a database that corresponds to a certain search. Concerning the overall quantity of relevant images kept in the repository, it is a performance statistic that evaluates how well the system can remember or retrieve the true positive occurrences, known as relevant images. Table 3 and Fig. 10 show the Recall of CBIR.

**Table 3:** Analysis of Recall.

Methods	Recall (%)
GLCM^32^	72
RULBP^32^	79
GLCM + DLECNN^32^	85
Xception^33^	92
MobileNet^33^	87
Inception^33^	92
CFTAB [proposed]	94

14 $$Recall=\frac{Tp}{Tp+Fp}$$

Compared with existing methods, the proposed method, CFTAB, achieves an impressive recall rate of 94%, outperforming GLCM (72%), RULBP (79%), and even GLCM + DLECNN (85%), Xception (92%), MobileNet (87%), Inception (92%). This indicates that CFTAB is capable of performing better in image retrieval and has the potential to be used in a wider range of applications.

### F1-score

**Fig. 11:**
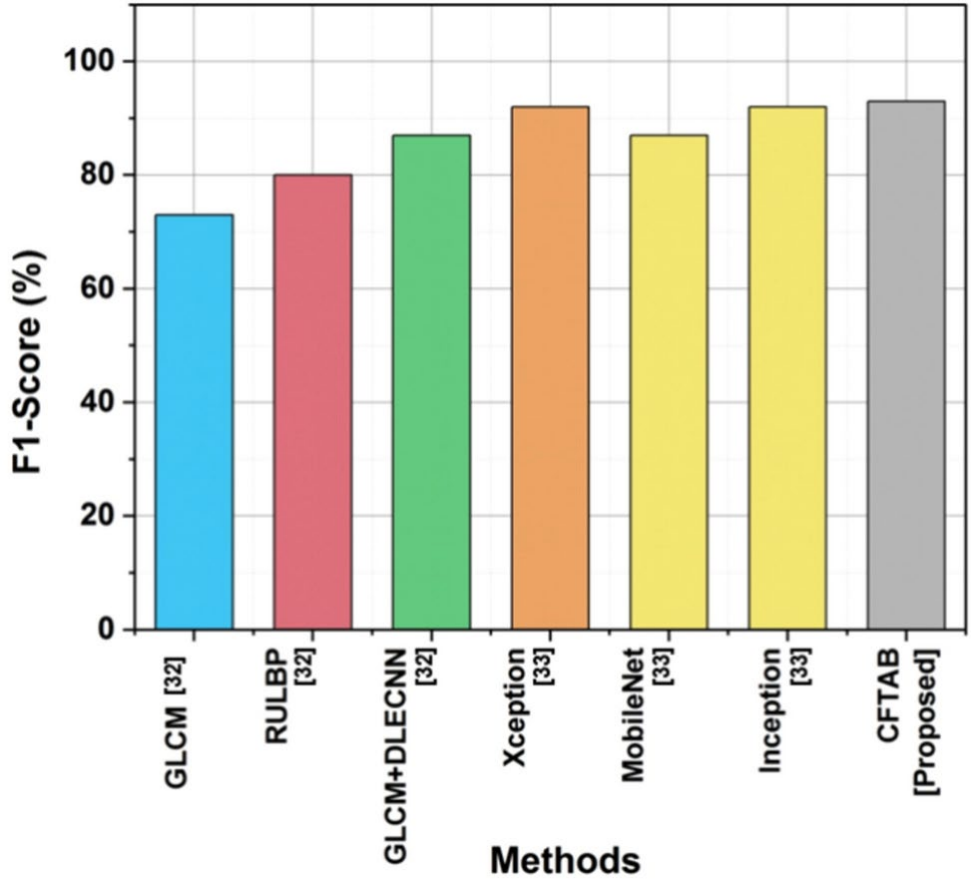
F1-score.

In CBIR, the F1-score is a statistic that is used to assess how well a system performs in locating relevant images. Precision and recall, two essential variables in information retrieval, are used to compute it. A higher number on the F1-score scale, which goes from 0 to 1, indicates a better balance between recall and accuracy. In the context of CBIR, a high F1 score indicates that the system minimizes the inclusion of irrelevant images while retrieving useful ones. Table 4 and Fig. 11 show the F1-score of CBIR.

**Table 4:** Outcome of F1-score.

Methods	F1-score (%)
GLCM^32^	73
RULBP^32^	80
GLCM + DLECNN^32^	87
Xception^33^	92
MobileNet^33^	87
Inception^33^	92
CFTAB [proposed]	93

15 $$F1 \; score=\frac{2\times Precision\times Recall}{Precision+Recall}$$

With an F1-Score of 93%, the suggested CFTAB approach achieves a remarkable performance in image analysis and retrieval compared to GLCM, RULBP, and GLCM + DLECNN, which scored 73%, 80%,925%,87%,92%, and 87%, respectively.

### Error rate

**Fig. 12:**
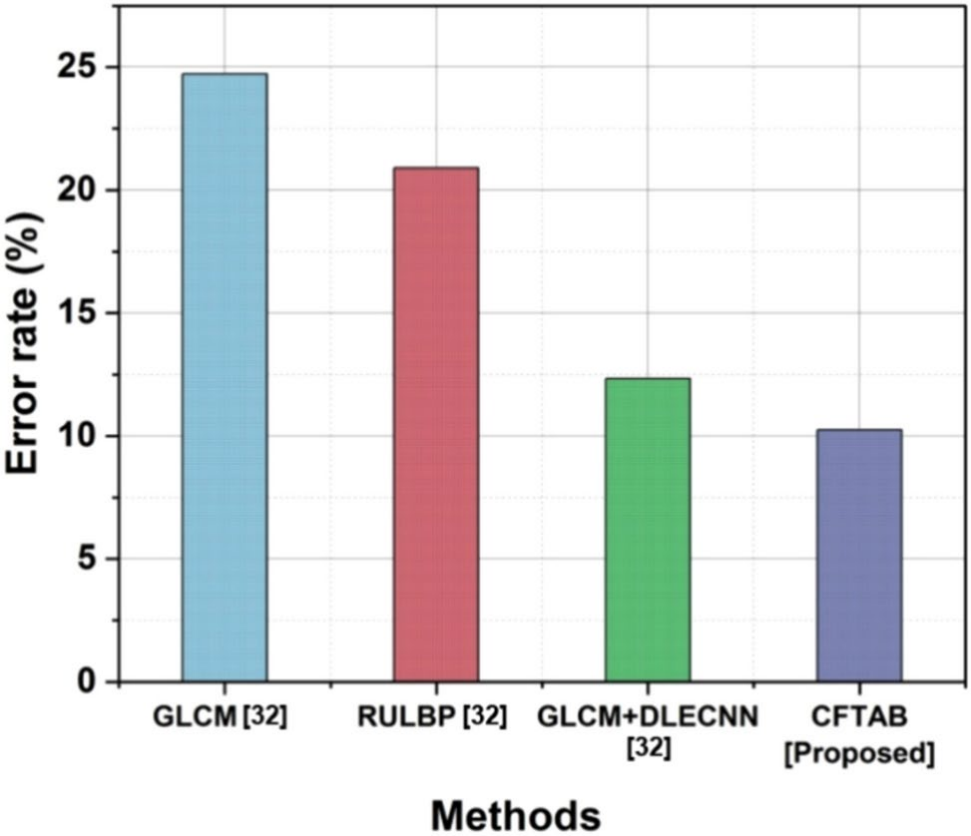
Error rate.

In CBIR the error rate measures the difference between the images that an image retrieval system that is focused on content finds the actual, relevant images. It’s computed as the relation of improperly obtained images to the searches or relevant images. Stated differently, it evaluates how well the system matches and retrieves images that are either visually or semantically related to the user’s query. Table 5 and Fig. 12 show the Error Rate of CBIR.

**Table 5:** Outcome of error rate.

Methods	Error rate (%)
GLCM^32^	24.73
RULBP^32^	20.9
GLCM + DLECNN^32^	12.33
CFTAB [proposed]	10.25

16 $$Error\;Rate=\frac{No. \; of\; Incorrectlly\; retrieved\; images}{total\;no\;of\;queries\;or\;relevant\; images\times 100}$$

As indicated in the corresponding study^30^, the suggested approach, CFTAB, shows a significant decrease in error rate at 10.25%, outperforming current methods like GLCM (24.73%), RULBP (20.9%) and GLCM + DLECNN (12.33%).

### Discussion

When compared to other deep learning algorithms like VGG19 and ResNet, image processing has advanced significantly from the days of GLCM, RULBP, and GLCM-DLECNN. More than capable of handmade feature extraction approaches; these contemporary designs use deep convolutional networks to automatically extract complicated hierarchical features. Xception^33^ while Xception performs well in terms of accuracy and efficiency by using depthwise separable convolutions, it can be computationally expensive due to its increased parameter count compared to simpler architectures like MobileNet. MobileNet^33^ addresses the computational limitations of Xception by using depthwise separable convolutions to reduce the number of parameters and operations. The Inception^33^ architecture, especially versions like Inception V3 and Inception V4, offers excellent performance in terms of accuracy by leveraging multi-scale feature extraction through inception modules. Among other things, VGG19 more effectively manages orientation variations and captures complex textures because to its deep layers and ResNet’s inventive skip relations. Their ability to achieve state-of-the-art performance in tasks like object identification and image classification, as well as their ability to simplify over a wide variety of datasets, demonstrate their strength in modern processor vision applications. These advancements show a paradigm shift in Favor of deep learning models, which excel in complex image analysis and offer solid solutions to practical issues.

## Conclusion

The CFTAB approach makes the process of generating CBIR simpler. The model is tailored to produce complicated visual attribute and trained using CNNs to achieve increased accuracy in rescue missions. By establishing ideal criteria, AB and others can further refine the classification process and achieve better recall and accuracy. Content-Based Image Retrieval (CBIR) systems are crucial in various applications, enabling users to explore and retrieve images based on their visual comfortable rather than relying solely on written metadata. The suggested revisions will enhance the effectiveness and efficiency of these systems. Combining these methods can improve the CBIR system’s feature extraction and decision boundary tuning, making it more sophisticated and effective overall. When compared to the cutting-edge models found in the CBIR dataset, the suggested strategy achieves greater results in terms of accuracy (97%), recall (94%), precision (95%), error rate (10.25%), and F1-score (93%). The approach is a major improvement over previous efforts in the field of image retrieval and approaches encouraging findings that highlight its potential for various image recognition, indexing, and retrieval applications.

Since the CNN network produces feature vectors as its output, it cannot be used to extract features from images alone to be relevant to the problem of accurately locating things by image. As a result of the items’ close size similarity, the model then searches using the K nearest neighbors, which produces unclear search results. To improve the deep neural network models’ accuracy, gather additional images and keep looking at other DL techniques to identify models with even better accuracy.

## Data Availability

The data presented in this study are available through email upon request to the corresponding author.
